# Society Meetings

**Published:** 1913-12

**Authors:** 


### SOCIETY MEETINGS

*Brief reports and announcements of meetings of societies of Western New York and of those of general scope are requested from Secretaries. Copy should be on hand by the fifteenth of each month. Full transactions will be published at cost of composition.*

---

The regular meeting of the Medical Society of the County of Erie was held in Alumni Hall, University of Buffalo, on Monday evening, October 20th, 1913.

President Whitwell called the meeting to order at 8.30 o'clock.

Secretary read the minutes of the regular meeting of June 16th, 1913, and also the minutes of the Council meeting of October 7th, 1913, both of which were approved as read.

Dr. Lytle, on behalf of the Committee on Membership, presented applications, which had been favorably acted upon by the Council, as follows:

Dr. Robert King, Buffalo State Hospital, and Dr. Christopher Fletcher, also located at the same institution, provided with certificates from the Secretary of the Medical Society of the County of St. Lawrence, showing that their dues were paid for the year 1913 and that they are in good standing in said society, were duly elected to membership in the Medical Society of the County of Erie.

Dr. Frank E. Brundage, 2561 Main street, coming from the Medical Society of the County of Allegany, and provided with a certificate from said society showing him to be in good standing and with dues paid for 1913, was duly elected by transfer.

Albert F. Ostwald, 1342 Fillmore avenue, Buffalo.

Dr. Nadina R. Kavincky, 576 Jefferson street, Buffalo, and Dr. Jennie Harper Harris, 160 Fletcher street, Tonawanda. N. Y., for whom the Secretary was directed to cast the ballot of the society, whereupon they were declared duly elected.

President Whitwell stated that nominations were now in order for the annual election, to be held on the third Monday in December, 1913.

The following nominations were then made:

Dr. John V. Woodruff for President.

Dr. Arthur W. Hurd for First Vice-President.

Dr. Franklin W. Barrows for Second Vice-President.

Dr. Franklin C. Gram for Secretary.

Dr. Albert T. Lytle for Treasurer.

For Censors, the present members of the Board of Censors, as follows:

Dr. John D. Bonnar, Dr. Francis E. Fronczak, Dr. Arthur G. Bennett, Dr. Irving W. Potter, Dr. Archibald D. Carpenter.

Dr. F. Park Lewis for Chairman of the Committee on Legislation.

Dr. Henry R. Hopkins nominated Dr. P. W. Van Peyma for Chairman of the Committee on Public Health. Dr. Van Peyma declined the nomination, stating that he would be unable to serve.

Dr. Grover W. Wende then nominated Dr. Henry R. Hopkins for such office.

Dr. Thomas H. McKee nominated Dr. Grover W. Wende for Chairman of the Committee on Membership.

The following were nominated as delegates to the State Society for the years 1914-15, four of whom are to be elected:

Dr. Albert T. Lytle, Dr. Julius Ullman, Dr. Julius Richter, Dr. J. F. Whitwell, Dr. LeLancey Rochester, Dr. Franklin W. Barrows.

Nominations were then declared closed.

Dr. Edith R. Hatch then presented a paper on the subject, "Is the Teaching of Sex Hygiene Advisable in Our Public Schools?"

This paper brought forth a very lively discussion for and against the proposition, by one of the largest attended meetings that the society has had for a long time.

There were present at this discussion, on invitation, Henry P. Emerson, Superintendent of Education, and a large number of other members of the department of education. At the close of the meeting a collation was served.

FRANKLIN C. GRAM, Secretary.

The regular meeting of the BUFFALO ACADEMY OF MEDICINE, Section of Medicine, was held in the Academy Rooms, Buffalo Public Library Building, on Tuesday evening, October 14th, 1913, at 8.30 P. M.

The program included a Symposium on the Proposed Ordinance for the Suppression of Unnecessary Noises. The speakers limited to five minutes each, were an aurist, a neurologist, a member of the Automobile Club, representatives of the Police Department, the Health Commissioner and others. Open discussion followed. The meeting was presided over by Dr. H. K. DeGroat, Chairman, and T. M. Leonard, Secretary.

After the reading of the minutes, Health Commissioner Francis E. Fronczak presented the following resolution for the purpose of opening a discussion on the unnecessary noises in the city of Buffalo, asking the Academy of Medicine to concur or amend it, and give the matter their moral support. The resolution follows:

"*Resolved,* That Section 10-A of Chapter XXV of the City Ordinances is hereby amended by adding the following:

No person, firm or corporation shall operate or cause to be operated, any machine, motor, engine, car, fan or other device which produces or causes any unnecessary noise or noises. And no person shall cause, create, produce or permit any person or anything in his or her control to produce any unnecessary noise, thereby disturbing the peace, quiet and comfort of persons in any neighborhood.

In all cases, the opinion of the Health Commissioner of the City, or his duly appointed agent, shall determine whether any given noise is unnecessary or is such as to disturb the quiet or comfort of persons in any neighborhood.

Any person guilty of making, causing or permitting any person or anything in his or her control to produce any unnecessary noise, thereby disturbing the peace, quiet, and comfort of any persons, shall be liable to the penalties mentioned in this Section."

The subject was fully discussed by Dr. Lucien Howe, who, from the point of an aurist, spoke on the influence on the nervous system through the ear splitting noises. Dr. Henry R. Hopkins, as Chairman of the Public Health Commission of the County Medical Society, Dr. DeLancey Rochester and Dr. Charles R. Jewett, from the general medical section, and Dr. J. W. Putnam from the standpoint of a neurologist; U. S. Thomas, representing the Automobile Club, spoke of the noises produced by the owners of automobiles and chauffeurs; Michael Regan, Superintendent of Police, presented a paper on the regulation of unnecessary noises by the police, calling special attention to the following Sections of Chapter 9: Sections 7, 9, 15, 19 and 20.

Section 7 reading as follows:

"Every person firing a cannon within the City, unless by permission of the Superintendent of Police, shall forfeit the penalty of $25.00; and there shall be no hunting or shooting with guns or firearms within the City unless by permission in writing of the Mayor of said City."

Section 9:

"No person shall ring any bell, blow any horn, make any public outcry, at or for any public sale, auction or vendue, or any private sale, or attract attention to or procure passengers for any cab, hack, hackney, coach or omnibus, in or upon any street, sidewalk or any other public place in the City."

Section 15:

"No person or persons shall, without permission of the Mayor, play upon any hand-organ, barrel-organ, barrel-accordion, barrel-piano, hurdy-gurdy, or other musical or wind instrument upon any street, sidewalk, crosswalk, dock, wharf, or any public ground within the City; nor take any part in or accompany any procession or company wherein such instruments, or any of them shall be played, or such singing, shouting or other such noise shall take place. This section shall not apply to military companies belonging to the National Guard, nor to regularly chartered civic or religious societies or orders, nor to funeral processions."

Section 19:

"No person, firm or corporation owning or controlling any engine or boiler, attached to which is a steam whistle, shall permit such whistle to be blown within the limits of the City, except upon vessels, crafts or floats in Buffalo Harbor and river and the waters connected therewith; nor shall any such vessel, craft or float use such whistle except for the purpose of giving the necessary marine signals. Any person, firm or corporation violating the provisions of this section shall be subject to a penalty of $10 for each and every offense."

Section 20:

"No person shall ring any church bell or other bell, when, on account of illness in the neighborhood, such ringing is forbidden by the Board of Health, under a penalty of $5 for each and every offense."

Speaking further, Superintendent Regan said: "No city can be absolutely noiseless. And the faster the city grows, the louder the noise, but there are many unnecessary noises about the city which should be stopped.

I don't believe that an employee of a railroad should be allowed to operate a whistle or allow steam to escape from a locomotive within the City unless for signal purposes. I believe the whistling is overdone.

The same applies, I believe, to boats. An ordinance should be passed prohibiting engineers from blowing whistles on tugs except for bridge and boat signals. The ordinance should be worded so that those arrested could not dodge the word 'signal.'

Railroad roadbeds, if properly laid, will prevent the awful noise and rattle on car lines which we have in some parts of the City. Then there is the unnecessary noise made by automobiles. Many have loud whistles and horns."

Health Commissioner Fronczak closed the discussion in a lengthy address, calling attention to the fact that in the nine different bureaus of the Department of Health, there are received annually tens of thousands of complaints of which a great number of them are against noises of various kinds, among them being the followings: Exhaust from gas and steam engines, exhaust or electric fans, flat car wheels, defective street car tracks, switches, uneven joints, etc.; machinery in bakeries, machinery in silk mills, drop and trip hammers, forging and boiler shops, machine shops, brewing machinery, manufacturers operating at night, roosters crowing, parrots on front verandas, steam whistles, church bells, automobile garages, automobile horns, whistles and exhausts, automobile manufacturers, the blowing of horns, piano playing, singing at night, the barking of dogs, cat fights, street carousing, New Year celebrating, practicing on band instruments, children skating on sidewalks, children running wagons, tricycles, velocipedes and other motion devices, children whistling and shouting, hammering on anvils, the squeaking of machinery, crying children, whistles on peanut roasters, etc., etc.

The proposed ordinance is for the purpose of putting all the complaints under one responsible authority, while at the present time they are divided between the Police, Street and Health Departments, Mayor's office, the Park Department, inspectors of boilers, the Common Council and still others, and there is decidedly too much of a division of responsibility.

Some of the city judges do not enforce the law, but on the contrary, give license to the offenders by calling them "dam fool ordinances." When the New Year is welcomed in at midnight, sometimes between fifty and seventy-five complaints come to the Health Commissioner personally, asking him to stop the unnecessary noises—whistles blowing, bells ringing, etc.

The entire subject matter was referred to the Council of the Academy to be considered before being presented at a stated meeting of the Academy.

---

Buffalo Academy of Medicine. October 25, a special meeting was held at the Buffalo Club. Prof. Dr. Adolph Schmidt of the University of Halle, presented a comprehensive paper on Hyperchlorhydria, which is promised for publication in this journal. A collation was served.

A stated meeting of the Academy was held November 5, at which Dr. John F. Fairbairn gave a paper on Acute Sinusitis, representing the Surgical Section. A committee was appointed to consider a place of meeting for the future, either in rented quarters or in a permanent home owned by the Academy, as might seem best.

The Medical Section met November 11, Drs. Eli H. Long and Karl F. Eschelmann demonstrating the use of the Sippy dilator on a case of cardiospasm of thirty years duration, and Dr. De Lancey Rochester reading a paper on Angina Pectoris, which is promised for publication in this journal.

The Section of Obstetrics and Gynæcology met November 18. Dr. F. C. Goldsborough showed a specimen of Perforation of Pregnant Uterus with Curette. Dr. W. Wayne Babcock of Philadelphia read a paper on Spinal Anæsthesia, Dr. Max C. Breuer leading the discussion.

---

The Medical Association of Central New York held its 45th annual meeting in Auburn, October 30, visiting members being entertained at luncheon by the Cayuga County Medical Society. The program was as follows:

1. President's Address—Louis F. O'Neill, M. D., Auburn.
2. The Treatment of Infantile Paralysis—Howard L. Prince, M. D., Rochester.
3. Tubercular Adenitis—George Price, M. D., Syracuse.
4. Treatment of Strangulated Hernia, with Report of Two Unusual Cases—Frederick Flaherty, M. D., Syracuse.
5. Injuries to Wrist Joint, Diagnosis and Treatment—C. E. Coon, M. D., Syracuse.

   Lunch.

   Business Session.
6. Medical Quackery in the Public Press—Samuel Hopkins Adams, Auburn.
7. The Purpose and Scope of the New State Health Law—William A. Howe, M. D., Phelps.
8. Diet in Health and Disease—Charles Clyde Sutter, M. D., Rochester.
9. The Choice of Anæsthetics with Special Reference to Local and Combination Anæthesia—Martin B. Tinker, M. D., Ithaca.
10. Hair Balls in the Stomach and Intestine—Ledra Heazlitt, M. D., Auburn.
11. The Present Status of Physical Therepeutics—A. H. Brown, M. D., Auburn.

Dr. M. Allen Richter was the essayist of the evening at the November meeting of the Medical Surgical League. His paper, entitled, "Office Dispensing; a Practical Demonstration," was a valuable lesson in pharmacy.

---

On November 19th, Col. Robert Henry Elliot, Supt. of the Government Ophthalmic Hospital at Madras, India, visited Buffalo as the guest of the Ophthalmological Club.

Col. Elliot has spent many years in ophthalmic service in India and has done more than all other surgeons combined to develop and popularize the operation of trephining the eye for the relief of glaucoma. Under Col. Elliot's leadership this operation is largely supplanting the older operation of iridectomy for the relief of intraocular tension, and from its very favorable results bids fair to become the operation of choice in the general surgical treatment of acute and chronic glaucoma.

Col. Elliot made his present visit to America at the invitation of the American Academy of Ophthalmology and Oto-Laryngology, and addressed a meeting of the Academy at Chattanooga last month on the subject of Sclero-Corneal Trephining.

On his visit to Buffalo he held a Surgical Clinic at the Buffalo General Hospital and demonstrated his operation on several cases of glaucoma. A large number of Ophthalmologists of Buffalo and Western New York were present at the Clinic.

In the evening the Buffalo Ophthalmological Club gave a dinner at the Saturn Club in honor of Col. Elliot.

---

The Hospital Medical Society of Rochester has the following program of meetings:

December 4—Results of Treatment in Rochester Open-Air School—John Aikman.

December 18—The Annual Meeting—William H. Sutherland.

---

The Rochester Pathological Society has the following program of meetings: November 13, Alvah C. Remington; November 28, William G. Bissell, Buffalo, N. Y.; December 11, Richard Moore; December 23, R. C. Harris.

---

The Monroe County Medical Society will hold its annual meeting December 16, 1913. There will be an election of officers and an address by Dr. C. R. Witherspoon, the retiring president.

---

The Rochester Academy of Medicine held its meeting on November 12, 1913, and Dr. F. C. Goldsborough of Buffalo read a paper on "Retroposition in the Puerperium." The next regular meeting is to be held on December 10, 1913.

The Blackwell Medical Society of Rochester has the following program of meetings: November 13, Dr. K. L. Daly on "Preventable Diseases"; December 11, Dr. M. May Allen, subject not announced.

